# Glial Tumor Necrosis Factor Alpha (TNFα) Generates Metaplastic Inhibition of Spinal Learning

**DOI:** 10.1371/journal.pone.0039751

**Published:** 2012-06-20

**Authors:** J. Russell Huie, Kyle M. Baumbauer, Kuan H. Lee, Jacqueline C. Bresnahan, Michael S. Beattie, Adam R. Ferguson, James W. Grau

**Affiliations:** 1 Department of Psychology, Texas A&M University, College Station, Texas, United States of America; 2 Brain and Spinal Injury Center, Department of Neurological Surgery, University of California, San Francisco, San Francisco, California, United States of America; Emory University, United States of America

## Abstract

Injury-induced overexpression of tumor necrosis factor alpha (TNFα) in the spinal cord can induce chronic neuroinflammation and excitotoxicity that ultimately undermines functional recovery. Here we investigate how TNFα might also act to upset spinal function by modulating spinal plasticity. Using a model of instrumental learning in the injured spinal cord, we have previously shown that peripheral intermittent stimulation can produce a plastic change in spinal plasticity (*metaplasticity*), resulting in the prolonged inhibition of spinal learning. We hypothesized that spinal metaplasticity may be mediated by TNFα. We found that intermittent stimulation increased protein levels in the spinal cord. Using intrathecal pharmacological manipulations, we showed TNFα to be both necessary and sufficient for the long-term inhibition of a spinal instrumental learning task. These effects were found to be dependent on glial production of TNFα and involved downstream alterations in calcium-permeable AMPA receptors. These findings suggest a crucial role for glial TNFα in undermining spinal learning, and demonstrate the therapeutic potential of inhibiting TNFα activity to rescue and restore adaptive spinal plasticity to the injured spinal cord. TNFα modulation represents a novel therapeutic target for improving rehabilitation after spinal cord injury.

## Introduction

The cytokine tumor necrosis factor alpha (TNFα) exerts a wide range of neuromodulatory effects, from promoting neuroprotection to inducing apoptosis and excitotoxic cell death [1], [2]. After spinal cord injury (SCI), TNFα activity has been implicated as a pathophysiological factor limiting behavioral recovery [3]. Overexpression of TNFα can induce neuropathic pain and exacerbate excitotoxicity [4], [5]. Recently, TNFα was shown to mediate early excitotoxic cell death following spinal contusion injury, and treatment with a TNFα inhibitor spared neurons and improved function across a range of behavioral outcome [6].

TNFα also affects neural plasticity [7]. TNFα modulates synaptic strength *in vitro* [8], [9] and TNFα overexpression in the hippocampus impairs spatial learning [10]. Recent work exploring the role of TNFα in spinal plasticity has focused on changes in nociceptive activity, showing that TNFα contributes to long-term potentiation (LTP) of C-fiber evoked field potentials, and plays a key role in the development of central sensitization [11], [12]. The capacity for the spinal cord to adapt and elicit altered behavioral outcomes in response to stimuli (adaptive spinal plasticity) is critical for functional recovery after injury, and understanding the neurobiological agents undermining adaptive plasticity will be essential for developing effective therapies [13], [14]. To assay spinal plasticity we used a high throughput *in vivo* assay of spinally-mediated learning [15]. In this preparation, electrical stimulation is delivered to the tibialis anterior muscle of a spinally-transected rat when the ankle is in an extended, unflexed position (controllable stimulation). Over time, the subject learns to keep the ankle flexed, minimizing stimulus exposure [15]. The spinal learning phenomenon provides an assay of adaptive spinal plasticity that predicts functional recovery after spinal contusion injury [16], [17]. Subjects that receive intermittent electrical stimulation independent of leg position (uncontrollable stimulation to either the contralateral leg or the tail) later fail to learn when tested with controllable stimulation [15]. Further work has shown intermittent stimulation has a lasting effect on the capacity for spinal learning and impairs recovery of function after spinal cord injury [17], [18].

We have shown that intermittent stimulation induces NMDA and metabotropic glutamate receptor activation, downstream protein kinase C activity, and requires *de novo* protein synthesis in order to inhibit spinal learning [19]–[21]. These findings suggest that intermittent stimulation is not inhibiting spinal learning by simply blocking plasticity in the spinal cord, but instead reflects an active process that modulates the capacity for subsequent plasticity (measured by the spinal learning outcome).

We have previously shown that this form of intermittent stimulation may undermine adaptive spinal learning by inducing diffuse alterations in nociceptive plasticity [22]. We have shown that intermittent stimulation produces bilateral tactile allodynia of the hindpaws [22], [23]. Conversely, we have recently demonstrated that other noxious stimuli that are known to produce inflammation, such as intradermal capsaicin or carrageenan, also undermine spinal instrumental learning [24]. Such findings suggest that intermittent stimulation may elicit a diffuse, central sensitization-like effect. Further, intermittent stimulation given acutely after contusion injury produces a long-term impairment in behavioral recovery [17]. Thus, peripheral noxious input (likely to accompany a natural spinal cord injury) may undermine functional recovery by inducing an inhibition of adaptive spinal plasticity. As uncontrolled nociceptive input is likely to accompany spinal cord injury [25], it will be essential to determine the biochemical mediators of maladaptive spinal plasticity in order to tailor treatments that not only attenuate maladaptive plasticity, but promote adaptive plasticity as well.

Here we tested the possible role of TNFα in the inhibition of adaptive spinal learning. We found that intermittent stimulation increases TNFα protein expression in the spinal cord. Intrathecal TNFα administration produced a long-term inhibition of spinal learning, and this effect required glial metabolism. Conversely, treatment with a TNFα inhibitor blocked the induction of the learning deficit produced by intermittent stimulation and rescued spinal learning if given after the deficit was induced by intermittent stimulation, TNFα, or a glial activator. Finally, we found the effects of both TNFα and intermittent stimulation appear to be mediated by calcium-permeable AMPA receptors, as treatment with a specific antagonist rescued the capacity for spinal learning. These findings highlight a critical role for TNFα in undermining adaptive spinal plasticity, and suggest that TNFα inhibitors may help rescue adaptive plasticity, providing a promising therapeutic avenue for functional rehabilitation following SCI.

## Methods

### Animals

All experiments were carried out in accordance with NIH standards for the care and use of laboratory animals (NIH publications No. 80-23), and were approved by the University Laboratory Animal Care Committee at Texas A&M University (AUP #2009-161).

Male Sprague-Dawley rats obtained from Harlan (Houston, TX) served as subjects. Rats were approximately 100–120 days old and weighed between 360 and 460 g. They were housed individually and maintained on a 12-hour light/dark cycle, with all behavioral testing performed during the light cycle. Food and water were available *ad libitum*.

### Surgery

Subjects were anesthetized with 5% isoflurane. The 2^nd^ thoracic vertebra (T2) was located by touch and a 2.5 cm anterior-posterior incision was made over T2. The tissue immediately rostral to T2 was cleared, exposing the spinal cord. A thermal cautery was then used to produce a complete transection of the cord, and the cavity was then filled with Gelfoam (Harvard Apparatus, Holliston, MA). A 25-cm polyethylene cannula (PE-10, VWR International, Bristol, CT) was subsequently threaded 9 cm down the vertebral column, into the subarachnoid space between the dura and the white matter so that the cannula lay on the dorsal surface of the spinal cord, over the L4-L5 spinal segments. The incision was closed using Michel clips (Fine Science Tools Foster, CA), and the exposed end of cannula tubing fixed to the skin with cyanoacrylate.

Immediately following surgery, subjects received an injection of 0.9% saline (2.5 mL, i.p.). During recovery, the hindlimbs were maintained in a normal flexed position using a piece of porous orthaletic tape, wrapped gently around the rat's body. The recovery period was 24 hours, throughout which the rats were housed in a temperature-regulated environment (25.5°C). Supplemental saline injections (0.9%, 2.5 mL, i.p.) were given daily to ensure proper hydration, and bladders expressed twice daily, and just before behavioral testing. Complete transections were confirmed by a) visually inspecting the cord during surgery, b) observing behavior following recovery, ensuring subjects exhibit paralysis caudal to the site of transection, and do not vocalize when shock is administered to the tail or hindpaw, c) examining the transection site postmortem in a randomly selected subset of subjects.

### Drug Administration

Rat recombinant TNFα (R&D Systems, Minneapolis, MN) was dissolved in phosphate-buffered saline (PBS) containing 0.1% bovine serum albumin (BSA) at a concentration of 60 or 600 pg/µL, delivered intrathecally in 10 µL. This dose response was based on our previous work showing that intraparenchymal injection of 60 or 600 pg TNF-induced AMPA receptor trafficking [6]. In the current experiments, the drug was administered intrathecally rather than by direct nanoinjection into the spinal cord, thus we increased the dose 10-fold to ensure bioavailability. Soluble TNF receptor 1 (sTNFR1, R&D Systems, Minneapolis, MN), which sequesters TNFα and reduces its endogenous activity, was dissolved in PBS containing 0.1% BSA at a concentration of 35 or 70 ng/µL, and delivered intrathecally in 10 µL. sTNFR1 concentration was determined by starting with 100th of the dose (3.5 µg) that has previously been found to attenuate neuropathic pain when given intrathecally [26]. TNFα and sTNFR1 were delivered by intrathecal injection in 10 µL. The glial metabolic inhibitor fluorocitrate (Sigma-Aldrich, St. Louis, MO) was dissolved in saline at a concentration of 4 nmol/µL and was delivered by intrathecal injection in 1 µL. Fluorocitrate has been previously shown to block the induction of a spinal learning deficit induced by intermittent stimulation [27]. Lipopolysachharide (LPS; Sigma-Aldrich, St. Louis, MO) was dissolved in saline at a concentration of 10 µg/µL, and delivered by intrathecal injection in 10 µL. This dose was chosen as it is been shown to induce nociceptive activity as well as produce a lasting spinal learning deficit [27], [28]. The calcium-permeable AMPA receptor antagonist 1- naphthyl acetyl spermine (Naspm) was dissolved in saline and given at a concentration of 10 mM in 10 µL. This dose has been shown to protect against ischemia-induced cell death in the hippocampus [29], [30]. All injections were followed by a 20 µL saline flush.

### Intermittent uncontrollable stimulation

**Figure 1:**
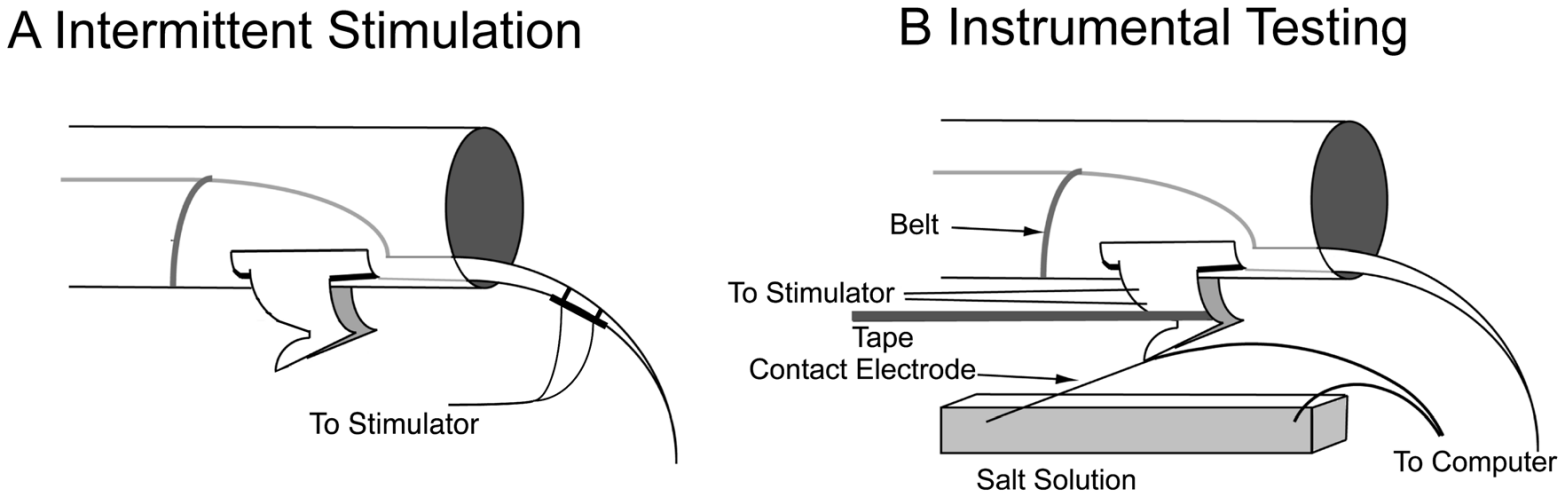
An overview of stimulation modes used to affect spinal plasticity. A) Intermittent Stimulation. Six minutes of peripheral electrical stimulation to the tail, that is not contingent upon leg position, is sufficient to induce a metaplastic inhibition of spinal instrumental learning. B) Instrumental Testing. To test spinal learning, spinally transected subjects are given electrical stimulation that is contingent upon hindpaw position. If the contact electrode is in contact with the salt solution, stimulation to the tibialis anterior muscle is delivered. Over time, subjects learn to keep the ankle in a flexed position, increasing response duration and reducing net stimulation exposure. If subjects are given intermittent stimulation prior to testing, spinal learning is inhibited.

Intermittent stimulation was administered while transected rats were loosely restrained in opaque black Plexiglas tubes, 22 cm in length and 6.8 cm in diameter. A flat floor constructed from a sheet of black Plexiglas 5.5 cm wide was attached 5.3 cm below the top of the tube. Electrical stimulation to the tail was delivered using an electrode constructed from a modified fuse clip. The electrode was coated with ECG gel (Harvard Apparatus, Holliston, MA) and secured with porous tape approximately 6 cm behind the base of the tail (Figure 1A). Constant-current 1.5-mA stimulation was delivered to the tail using a 660-V transformer. A Macintosh computer controlled the onset and offset of stimulation. Each stimulation was 1.5 mA in intensity, 80 ms in duration, delivered over the course of 6 minutes. The stimuli were delivered intermittently in a randomized fashion between 0.2 and 3.8 seconds apart. Subjects received a single 6-minute session, over which 180 shocks were presented, at an average of 2 seconds apart.

### ELISA

TNFα levels were assessed using the TNFα immunoassay kit from R&D Systems (Minneapolis, MN). Briefly, subjects were deeply anesthetized with pentobarbital (50 mg/kg, i.p.) at one of three timepoints: 20 minutes, 6 hours, or 24 hours after cessation of stimulation. Spinal cords were harvested, flash-frozen in liquid nitrogen, and stored at −80°C. Spinal tissue samples (1 cm in length, L4-S2 spinal segments) were homogenized in cold lysis buffer (phosphate-buffered saline, pH 7.4, with 1% Triton-X 100 and Roche Minicomplete protease inhibitor cocktail). Supernatants were obtained by centrifugation (13,000 g for 15 min at 2°C) and stored at −80°C until assays were conducted according to kit instructions. Absorbance was measured on a Victor 2 microplate reader (Perkin Elmer/Cetus, Norwalk, CT) and TNFα concentrations were normalized to total protein determined with the bicinchoninic acid (BCA) method.

### Instrumental testing procedure

All subjects were allowed to recover for at least 24 h following surgery and the hindlimbs were shaved and marked for electrode placement prior to testing. Instrumental testing was conducted while rats were loosely restrained in tubes (23.5 cm [length]×8 cm [internal diameter]) (Figure 1B). Two slots in the tube, (5.6 cm [length]×1.8 cm [width]), 4 cm apart, 1.5 cm from the end of the tube, allowed both hind legs to hang freely. To minimize the effects of upper body movement on leg position, a wire belt was secured to the rat's trunk within the tube. Leg stimulation was delivered using a BRS/LVE (Laurel, MD) constant current (60 Hz, AC) shock generator (Model SG-903).

A wire electrode was inserted through the skin over the distal portion of the tibialis anterior (1.5 cm from the plantar surface of the foot), and one lead from the generator was attached to this wire. A contact electrode (7 cm in length, 0.46 diameter, stainless steel) was secured to the foot between the second and third digits with a piece of porous tape. A fine wire (0.01 sq mm [36 AWG] (20 cm) attached to the end of the contact electrode extended from the rear of the foot and connected to a digital input monitored by a Macintosh computer. A plastic rectangular dish (11.5 [w]×19 [l]×5 [d]) containing a salt solution was placed approximately 7.5 cm below the restraining tube. A drop of soap was added to the solution to reduce surface tension. A ground wire was connected to a 1 mm wide stainless steel rod, which was placed in the solution. The shock generator was set to deliver a 0.4 mA shock, and the proximal portion of the tibialis anterior (approximately 1.7 cm proximal to the wire electrode) was probed with a 2.5-cm stainless steel pin attached to a shock lead to find a robust flexion response. The pin was then inserted 0.4 cm into the muscle. A strain gauge was utilized to determine the amount of shock necessary to elicit a 0.4 N flexion force, as this amount of force has been shown previously to be ideal to produce the flexion necessary for spinal instrumental learning [15].

To minimize lateral leg movements, a 20-cm piece of porous tape was wrapped around the leg and attached to a bar extending across the apparatus directly under the front panel of the restraining tube. The tape was adjusted so that it was taut enough to slightly extend the knee. Finally, three short (0.15 s) shock pulses were applied and the level of the salt solution was adjusted so that the tip of the contact electrode (attached to the rat's foot) was submerged 4 mm below the surface.

A rat's capacity to perform the instrumental response was then tested with exposure to 30 min of controllable shock. Whenever the rat's leg fell below the level of the salt solution, the electrodes delivered a shock to the tibialis anterior muscle causing the ankle to flex. Leg position was monitored using a Macintosh computer at a sampling rate of 30 Hz.

### Behavioral measures

Three behavioral measures, response number, response duration and time in solution, were used to assess a subject's capacity to perform the instrumental response [15]. Performance was measured over time in 30 1-min time bins. The computer monitoring leg position recorded an increase in response number whenever the contact electrode was raised above the salt solution. Response duration was derived from time in solution and response number using the following equation: Response Duration_i_ = (60 s−time in solution_i_)/(Response Number_i_+1) where i is the current time bin.

### Statistical Analyses

The ELISA experiment was cross-sectional; each post-stimulation timepoint for spinal tissue collection was performed as an individual experiment. As such, planned-comparisons were used to compare unstimulated and intermittently-stimulated subjects at each timepoint. All spinal learning experiments were run with a balanced, full-factorial design and analyzed using mixed ANOVAs with an *a priori* alpha value of *p*<0.05 considered significant. This design allowed us to test the overall main effects of drug/stimulation condition and time, as well as interactions between these variables with a single general linear model (SPSS-GLM, v.19). Significant interactions were followed up with one-way ANOVAs on group means, which allowed for *post hoc* testing (Duncan's New Multiple Range). This analytic approach is consistent with well-established standards within the statistical literature [31].

## Results

### Intermittent stimulation increases TNFα protein expression

To investigate the dynamics of stimulus-induced TNFα expression, we assessed spinal TNFα protein expression after intermittent stimulation. Although stimulation to the tail directly drives sensory afferents in the sacral-coccygeal region, previous work has shown that this stimulation also produces a diffuse effect on more rostral spinal segments, as evidenced by the capacity for intermittent tail stimulation to inhibit spinal learning, as well as produce a tactile allodynia in the hindpaws [18], [22], [23]. As we have previously shown that the essential anatomical locus for the expression of the spinal learning effect lies in the L4-S2 region, TNF protein expression was assessed in spinal cord blocks from this region [32].

Spinally-transected rat subjects (n = 6 subjects per group) received either 6 minutes of intermittent stimulation or an equivalent period of unstimulated restraint. Subjects were then deeply anesthetized with pentobarbital (50 mg/kg, i.p.) at one of three timepoints after treatment: 20 minutes, 6 hours, or 24 hours. As before, spinal cord sections (L4-S2) were removed, flash-frozen with liquid nitrogen, and stored at −80°C. Tissue was then subsequently homogenized and processed for assessment with enzyme-linked immunosorbent assay (ELISA) as described in the Methods section.

**Figure 2:**
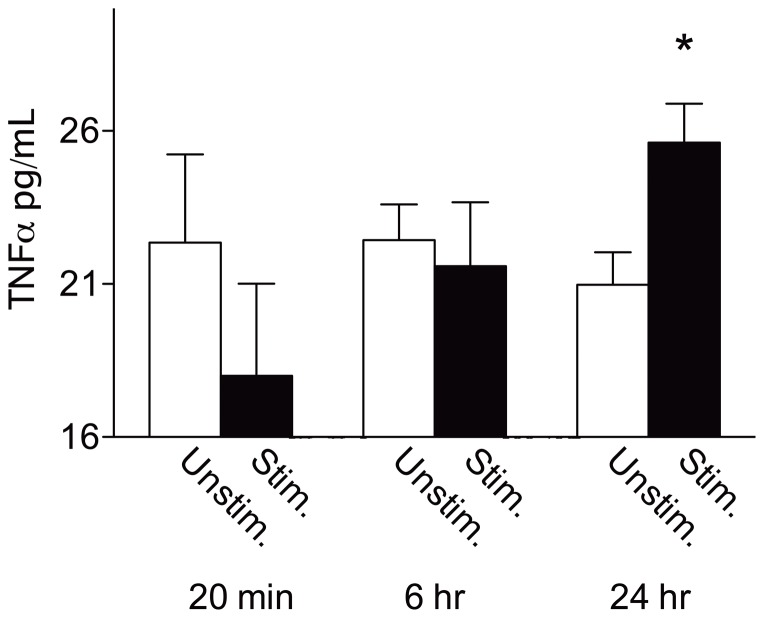
Intermittent stimulation increases TNFα protein expression. ELISA data are presented as mean TNFα protein expression; variability is expressed as SEM. Intermittent stimulation produced an increase in TNFα protein culminating in a significant increase over unstimulated control subjects at 24 hours. Asterisk signifies statistical significance, *p*<.05.

The effect of intermittent stimulation on TNFα protein expression is depicted in Figure 2. TNFα protein expression was unchanged for unstimulated controls across timepoints. Planned comparisons were performed at each time point revealing that TNFα protein expression after intermittent stimulation was significant at 24 hours [F_(1, 10)_ = 6.49, *p*<.05], but not at 20 minutes or 6 hours.

### Intermittent stimulation-induced inhibition of spinal learning requires endogenous TNFα activity

As the previous experiments showed that intermittent stimulation can induce an increase in TNFα protein expression, the current experiment was designed to test whether endogenous TNFα activity is necessary in order for intermittent stimulation to undermine future spinal learning. Twenty-four hours after complete transection, subjects (n = 8 per group) received an intrathecal injection of either soluble TNF receptor sTNFR1 (350 ng), which acts to inhibit TNFα activity by sequestering endogenous soluble TNFα, or vehicle (PBS+0.1% BSA). Forty-five minutes later, subjects received either 6 minutes of intermittent stimulation or an equivalent period of unstimulated restraint. All subjects were then immediately tested for spinal instrumental learning.

**Figure 3:**
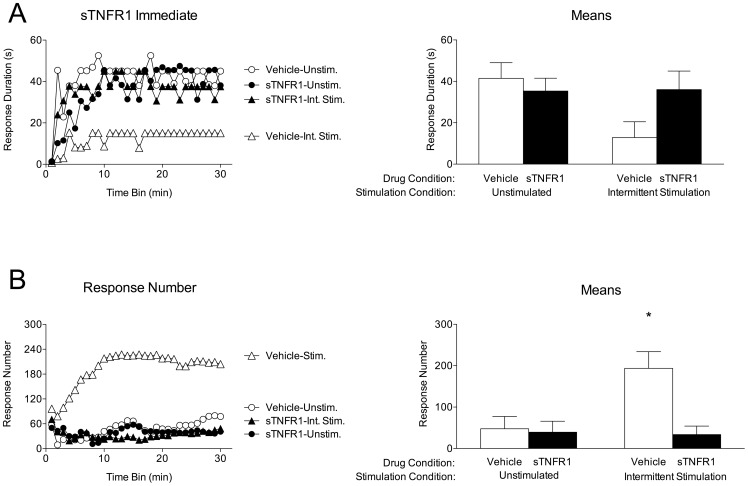
Blocking endogenous TNFα activity prior to intermittent stimulation protects against the inhibition of spinal learning. Subjects received intrathecal injection of either 350 ng of the TNFα inhibitor sTNFR1 or vehicle, followed by 6 minutes of intermittent electrical stimulation (Stimulated) or none (Unstimulated). All subjects were then immediately tested for instrumental learning. A) Left panel depicts response durations over time. Increased response durations over time are indicative of spinal instrumental learning. Right panel depicts mean response durations for each group, collapsed over time. Error bars represent SEM for group means. B) Left panel depicts response number over time. Right panel depicts mean response number for each group. Data show that subjects who failed to learn had higher response numbers, confirming that these subjects are not exhibiting a deficit in response performance. Shaded areas represent SEM over time; error bars represent SEM for group means.

As expected, vehicle-treated unstimulated subjects exhibited an increase in response duration over the testing session, indicative of spinal learning (Fig. 3A). Likewise, subjects that received the TNFα inhibitor sTNFR1 alone were able to learn. Vehicle-treated subjects that received intermittent stimulation exhibited a pronounced learning deficit. Interestingly, those subjects that received the TNFα inhibitor prior to intermittent stimulation exhibited no learning deficit. An ANOVA revealed a main effect of time [F_(29,812)_ = 7.58] and a significant three-way interaction between time, drug, and stimulation, F_(29, 812)_ = 1.54, *p*<.05. This interaction indicates that learning (the adaptive change in flexion duration over time) is dependent on *both* stimulation condition and TNFα inhibitor treatment. No other effects were significant, *p*>.05.

To evaluate whether our experimental treatment affected baseline behavioral reactivity, we analyzed both the shock intensity required to elicit a flexion force of 0.4 N and the duration of the first shock-elicited flexion response. Independent analyses of variance (ANOVAs) showed that there were no group differences on either measure, *F*s<2.58, *p*<0.05. We found no significant differences on these measures in any of the subsequent spinal learning experiments, therefore those data are not shown.

The number of responses made by each subject was also assessed (Figure 3B). On average, subjects that exhibited the learning deficit exhibited the highest rate of responding, while those that learned responded less frequently. The difference in total response number was assessed using an ANOVA, revealing main effects of drug [F_(1, 28)_ = 6.81], stimulation [F_(1, 28)_ = 4.74], and time [F_(29, 812)_ = 3.82], as well as significant Drug X Stimulation [F_(1, 28)_ = 5.53], Time X Drug [F_(29, 812)_ = 3.90], and Time X Drug X Stimulation interactions [F_(29, 812)_ = 2.02], *p*<.05. *Post hoc* analysis of group means showed that vehicle-treated subjects receiving intermittent stimulation were significantly different from all other groups, *p*<.05. This pattern of results is similar to that reported in prior studies [15], [18] and emerges because rats that fail to learn respond in a mechanical manner to stimulation. For these subjects, stimulation elicits a flexion response, but does not produce an increase in flexion duration (our index of learning). This observation is important because it demonstrates that the inhibition of learning does not reflect an inability to perform the target response (i.e., a performance deficit). Because all subsequent experiments yielded a similar inverse relationship between response duration and response number, and because the former measure provides an index of learning that avoids some interpretative problems [15], we only report response duration in subsequent experiments.

### Endogenous TNFα affects both induction and expression of the stimulation-induced inhibition of spinal learning

The prior experiment showed that pretreatment with a TNFα inhibitor prior to intermittent stimulation blocked the learning deficit when subjects were tested immediately, i.e. while the drug was still effective. Expanding on that finding, the current experiment examined the long-term effect of a TNFα inhibitor when given either prior to intermittent stimulation or prior to testing. In this way, the current experiment tests whether endogenous TNFα is necessary for intermittent stimulation to *induce* the spinal learning deficit, as well as tests the possibility that TNFα is necessary in order for the learning deficit to be *expressed* following intermittent stimulation.

**Figure 4:**
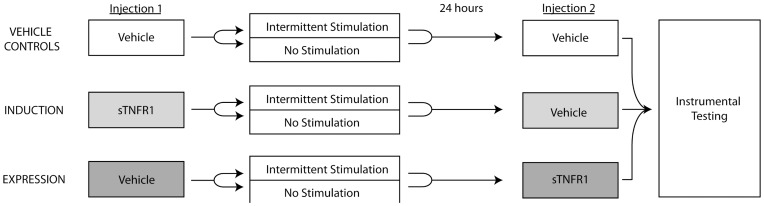
Experimental design for Experiment 2. Twenty-four hours after complete transection, all subjects were given a first injection of either sTNFR1 (350 ng) or vehicle, followed by either intermittent stimulation or nothing, and then 24 hours later, received a second injection of either sTNFR1 (350 ng) or vehicle. All subjects were then tested for instrumental learning. Subjects were divided into three groups; Control, in which they received vehicle at both injection timepoints; Induction, in which they received sTNFR1 *prior* to intermittent stimulation (or no stimulation); and Expression, in which they received sTNFR1 24 hours *after* intermittent stimulation (or no stimulation).

The design of this experiment is depicted in Figure 4. All subjects were given complete transections at T2 24 hours before experimental manipulation began. To test the long-term effect of intermittent stimulation, two groups (n = 8 subjects per group) were given vehicle injections, followed 45 minutes later with either 6 minutes of intermittent stimulation or an equivalent period of unstimulated restraint. Twenty-four hours later, subjects were given a second vehicle injection, followed by instrumental testing. To test the effect of TNFα inhibition on the induction of the spinal learning deficit, two groups (n = 8 subjects per group) were given sTNFR1 (350 ng), followed 45 minutes later with 6 minutes of intermittent stimulation or an equivalent period of unstimulated restraint. Twenty-four hours later, subjects were given a vehicle injection, followed by instrumental testing. Finally, to test the effect of TNFα inhibition on the expression of the learning deficit, two groups (n = 8 subjects per group) were first given vehicle injections, followed 45 minutes later with 6 minutes of intermittent stimulation or an equivalent period of unstimulated restraint. Twenty-four hours later, subjects received an intrathecal injection of sTNFR1, followed by instrumental testing. This design allowed us to: a) verify that treatment with intermittent stimulation inhibits spinal learning 24 hours later, b) test whether sTNFR1 *before* intermittent stimulation blocks the *induction* of the learning deficit, and c) test whether sTNFR1 *after* intermittent stimulation blocks the *expression* of the learning deficit. The design of this experiment allowed for the same stimulated and unstimulated vehicle-treated controls to be compared to both the induction and expression groups in the subsequent analyses.

**Figure 5:**
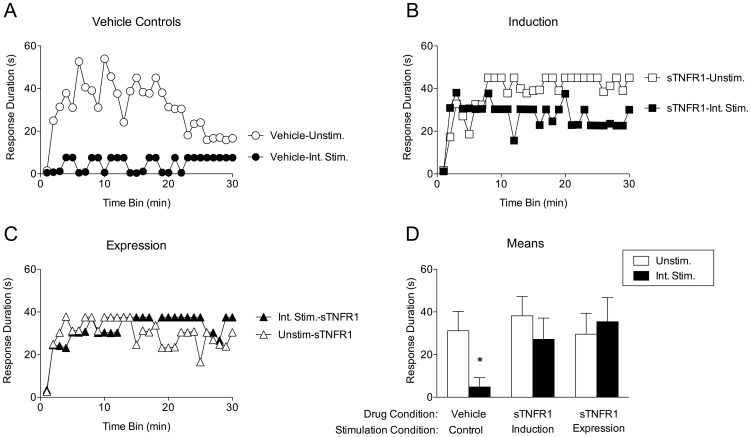
Inhibition of endogenous TNFα blocks the induction and expression of the inhibition of spinal learning. A) Vehicle Controls. Response durations increased over time for those that did not receive intermittent stimulation (Unshocked), while those that did receive intermittent stimulation (Shocked) exhibited a learning deficit. These groups were used to compare to the Induction and Expression experimental groups. B) Induction. Response durations increased over time for both groups, indicating that sTNFR1 blocked intermittent stimulation from inducing a spinal learning deficit. C) Expression. Response durations increased over time for both groups, indicating that sTNFR1 blocked the expression of the intermittent stimulation-induced learning deficit. D) Group means collapsed over time. The group that received intermittent stimulation in the absence of sTNFR1 had significantly lower response durations over time than all other groups. Shaded areas represent SEM over time; error bars indicate SEM for group means. Asterisk signifies statistical significance, *p*<.05.

As expected, prior exposure to intermittent stimulation inhibited spinal learning in vehicle-treated subjects (Figure 5A). The effect of TNFα inhibition on the *induction* of the stimulus-induced learning deficit is depicted in Figure 5B. Subjects that received the TNFα inhibitor prior to intermittent stimulation exhibited no learning deficit. An ANOVA comparing these subjects to the vehicle controls revealed main effects of drug [F_(1, 28)_ = 4.24], stimulation condition [F_(1,28)_ = 6.84], and time [F_(29, 812)_ = 3.45], *p*<.05. Further, the Time X Stimulation interaction [F_(29, 812)_ = 1.67] and the Time X Drug X Stimulation interaction [F_(29, 812)_ = 2.38] were both significant, *p*<.05. *Post hoc* comparison of the group means confirmed that the vehicle-treated intermittent stimulated group differed significantly from all other groups, *p*<.05. No other effects were significant, *p*>.05.

The effect of TNFα inhibition on the *expression* of the stimulus-induced spinal learning deficit is depicted in Figure 5C. As in the induction groups, those that received sTNFR1 alone had no impairment in learning. Surprisingly, those that received sTNFR1 following intermittent stimulation were also able to learn. An ANOVA comparing these groups to the vehicle controls revealed a significant main effect of trials [F_(29, 812)_ = 3.51], as well as a significant Time X Stimulation interaction, [F_(29, 812)_ = 2.30], *p*<.01. *Post hoc* comparison of the group means confirmed that the vehicle –treated group that received intermittent stimulation differed significantly from all other groups, *p*<.05. No other effects were significant, *p*>.05.

The protective effect of TNFα inhibition found in the induction groups of this experiment extends the findings from the previous experiment, in which sTNFR1 was shown to protect against the stimulation-induced learning deficit when subjects were tested immediately following intermittent stimulation. Here, this protective effect was evident when subjects were tested 24 hours after intermittent stimulation.

Beyond the protective effect of TNFα inhibition, this experiment also demonstrates that sTNFR1 can provide a therapeutic effect, blocking the expression of the stimulation-induced learning deficit when given 24 hours after intermittent stimulation. That learning can be rescued by blocking TNFα activity suggests that the role for TNFα in the inhibition of spinal learning is not a transient one; rather, the capacity for TNFα inhibition to restore learning long after the deficit has been induced suggests that intermittent stimulation may not only cause TNFα release, but that this release may be sustained.

### Spinal administration of exogenous TNFα generates a long-term inhibition of spinal learning

It has been shown that the overexpression of TNFα can lead to a robust increase in neural excitability that ultimately undermines learning [10]. Similarly, a number of pharmacological agents that are known to induce the spinal learning deficit have been correlated with increased TNFα release [33]–[35]. Here we tested whether administration of exogenous TNFα can substitute for intermittent stimulation to produce a spinal learning deficit.

Twenty-four hours after complete transection, subjects (n = 8 per group) received an intrathecal injection of one of two doses of TNFα (600 or 6000 pg) or vehicle (PBS+0.1% BSA). Subjects were then tested for instrumental learning either 45 minutes after injection, or 24 hours after injection.

**Figure 6:**
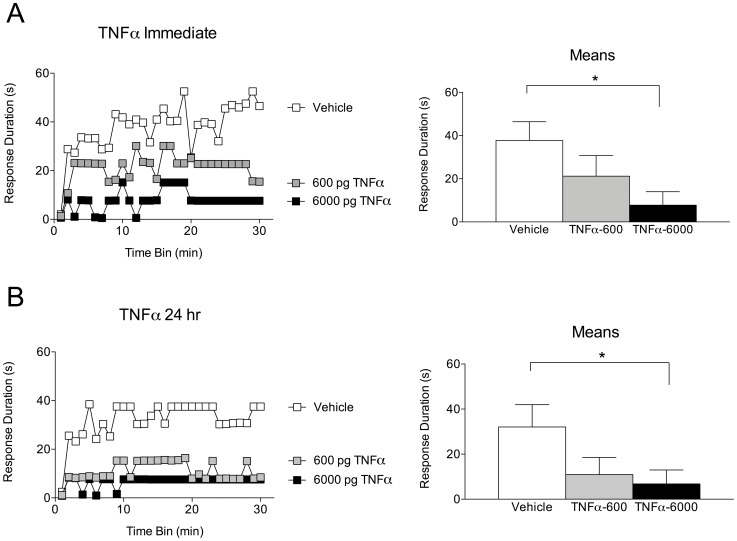
Administration of exogenous TNFα generates immediate and lasting metaplastic inhibition of spinal learning. A) Subjects received an intrathecal injection of 600 pg TNFα, 6000 pg TNFα, or vehicle. All groups were then tested for spinal instrumental learning. Left panel depicts response durations over time. TNFα administration undermined spinal learning in a dose-dependent fashion. Shaded area represents SEM over time. Right panel shows group response duration means collapsed over time. The group that received the highest dose of TNFα differed significantly from the vehicle-treated group. Error bars represent SEM for the group means. Asterisk signifies statistical significance, *p*<.05. B) Subjects received an intrathecal injection of 600 pg TNFα, 6000 pg TNFα, or vehicle 24 hours prior to instrumental testing. Left panel depicts response durations over time. TNFα administration undermined spinal learning in a dose-dependent fashion. Right panel shows group response duration means collapsed over time. The group that received the highest dose of TNFα differed significantly from the vehicle-treated group. Shaded areas represent SEM over time; error bars indicate SEM for group means. Asterisk signifies statistical significance, *p*<.05.

As expected, vehicle-treated subjects were able to learn (Figure 6). Those that received the highest dose of TNFα (6000 pg), either 45 minutes or 24 hours prior to testing, exhibited a learning deficit (Figure 6A & B). An ANOVA revealed main effects of drug treatment [F_(2, 42)_ = 6.96] and time [F_(29, 1218)_ = 4.78], as well as a significant Drug X Time interaction [F_(58, 1218)_ = 1.38], *p*<.05. *Post hoc* analyses of the group means also showed a significant difference between the highest dose of TNFα and vehicle, at both the immediate and 24 hour time points, *p*<.05. No other effects were significant, *p*>.05.

The previous two experiments demonstrated that endogenous TNFα was necessary to produce a stimulus-induced spinal learning deficit. Here we expand on those findings by showing that administration of TNFα is also sufficient to produce both acute and long-term inhibition of spinal learning. The highest doses tested at either timepoint caused a deficit that is commensurate with the level of impairment seen in intermittently-stimulated subjects, lending further evidence that TNFα activity may mediate the stimulus-induced inhibition of spinal learning.

### TNFα inhibition prior to testing restores the capacity for spinal learning

Prior work has shown that TNFα administration can lead to an increased expression of endogenous TNFα stores [36]. Such sustained TNFα activity could mediate the long-term TNFα-induced learning deficit that was observed in the previous experiment. The current experiment was designed to address this possibility, assessing whether the long-term spinal learning deficit induced by exogenous TNFα can be blocked by inhibiting TNFα activity prior to testing.

Twenty-four hours after complete transection, subjects (n = 8 per group) received an intrathecal injection of TNFα (6000 pg) or vehicle (PBS+0.1% BSA). Twenty-fours later, subjects were given the TNFα inhibitor sTNFR1 or vehicle 45 minutes prior to testing. Because pilot data indicated a 350 ng dose of sTNFR1 had a partial effect, two additional groups were added that were treated with a higher dose (700 ng) of sTNFR1 prior to testing.

**Figure 7:**
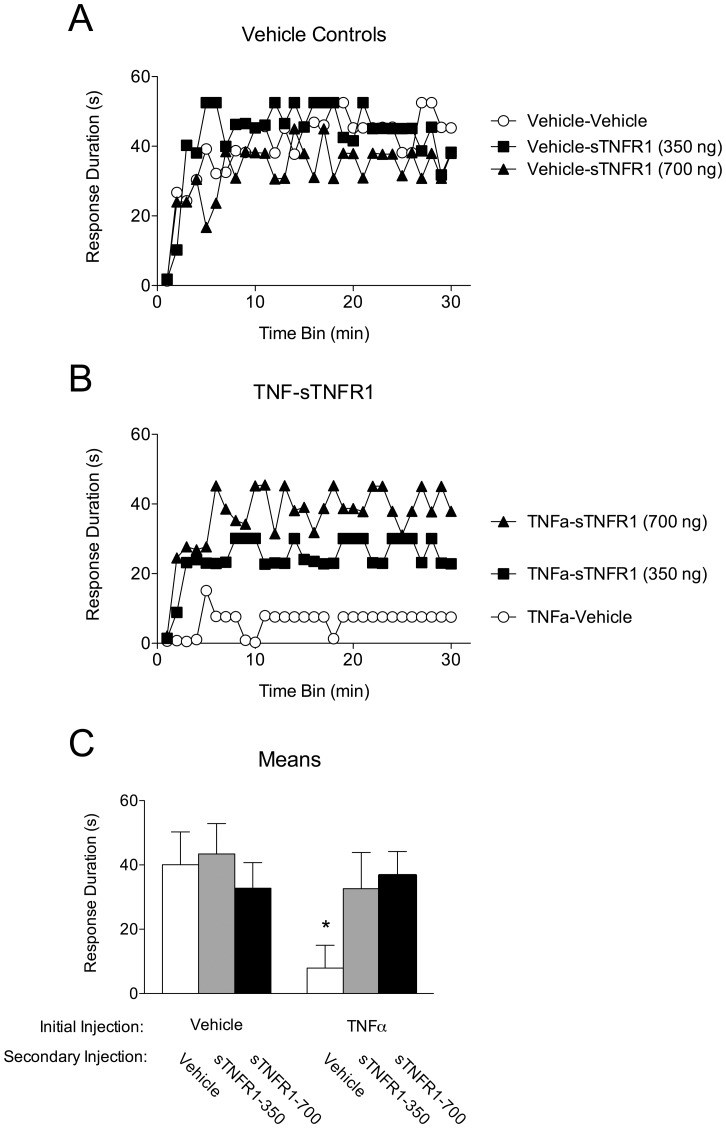
TNFα inhibition reverses the lasting inhibition of spinal learning induced by TNFα. Subjects received an intrathecal injection of TNFα (6000 pg) or vehicle, followed 24 hours later by an injection of 350 ng sTNFR1, 700 ng sTNFR1, or vehicle. All subjects were then tested for instrumental learning. A) Vehicle controls. Subjects that did not receive a TNFα injection exhibited increased response durations over time, indicative of spinal instrumental learning. B) Of those that received TNFα injections, those treated with either 350 ng or 700 ng sTNFR1 24 hours later were able to exhibit an increase in response duration over time. C) Group response duration means collapsed over time. Those that received TNFα alone had significantly lower response durations than all other groups. Shaded areas represent SEM over time; error bars indicate SEM for group means. Asterisk signifies statistical significance, *p*<.05.

As expected, subjects that received only vehicle injections were able to learn (Figure 7A). Vehicle-treated subjects that received sTNFR1, at either dose, also learned. Subjects given TNFα alone exhibited a learning deficit when tested 24 hours later, replicating the finding from the previous experiment (Figure 7B). Interestingly, TNFα-treated subjects that were given sTNFR1 prior to testing exhibited no learning deficit. An ANOVA revealed a significant main effect of both TNFα treatment and time, F>6.75, *p*<.05. Although no other main effects or interactions were significant, the interaction between TNFα treatment and sTNFR1 treatment approached significance, F _(2,42)_ = 3.13, *p* = .054. To further explore this relationship, trend analyses were run. These analyses revealed that the linear component of the Time X TNF X sTNFR1 interaction was significant, F = 10.42, *p*<.01. The trend analyses also showed that the quadratic component of the Time X sTNFR1 interaction to be significant, F = 5.81, *p*<.05. Finally, a *post hoc* comparison of the group means revealed that the group that received TNFα alone differed significantly from all other groups, *p*<.05 (Figure 7C). This finding suggests that TNFα activity is still required 24 hours after TNFα treatment in order for the learning deficit to be expressed, and lends evidence for the possibility that exogenous TNFα administration may elicit an increased expression of endogenous TNFα that outlasts the initial treatment.

### Inhibition of glial metabolism blocks TNF-induced inhibition of spinal learning

TNFα is known to be released primarily from glial cells. Kuno et al. (2005) demonstrated that administration of exogenous TNFα to cultured microglia could induce the sustained production and release of endogenous TNFα [36]. Thus, if TNFα underlies the long-term effect of intermittent stimulation by inducing further TNFα expression, then the maintenance (memory) of that effect should depend on glial activity. Supporting this, Vichaya et al. (2009) have shown that the glial metabolic inhibitor fluorocitrate given prior to intermittent stimulation blocked the induction of the spinal learning deficit [27]. The current experiment explored whether the administration of fluorocitrate prior to TNFα treatment would block the long-term effect of TNFα on spinal learning.

Twenty-four hours after complete transection, subjects (n = 6 per group) received an intrathecal injection of either the glial inhibitor fluorocitrate (4 nmol) or saline vehicle. Forty-five minutes later, subjects received TNFα (6000 pg) or vehicle. All subjects were then tested for instrumental learning 24 hours later.

**Figure 8:**
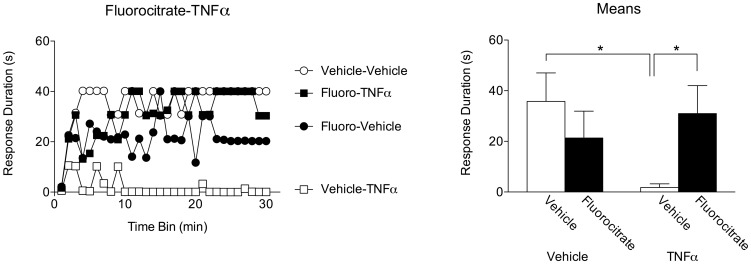
TNF-induced inhibition of spinal learning requires glial metabolism. Subjects received an intrathecal injection of either the glial metabolic inhibitor fluorocitrate (4 nmol) or vehicle, followed by either TNFα (6000 pg) or vehicle. All subjects were then tested for instrumental learning 24 hours later. Left panel depicts response durations over time. Those that did not receive TNFα were able to exhibit increased response durations over time. Those that received fluorocitrate prior to TNFα were also able to learn. Right panel shows group response durations collapsed over time. The group that received TNFα alone differed significantly from vehicle controls, as well those that received fluorocitrate prior to TNFα. Shaded areas represent SEM over time; error bars indicate SEM for group means. Asterisk signifies statistical significance, *p*<.05.

Subjects that only received vehicle treatment were able to learn as expected (Figure 8). Likewise, those subjects that received fluorocitrate alone also learned, replicating prior findings [27]. Subjects receiving TNFα alone exhibited a marked learning deficit, and interestingly, this effect was blocked in subjects that received fluorocitrate prior to TNFα treatment. An ANOVA revealed a significant interaction between drug treatments, F_(1,20)_ = 6.30, *p*<.05. There was a significant main effect of time [F_(29, 580)_ = 2.00], as well as a significant interaction between time, TNFα, and fluorocitrate treatment, F_(29, 580)_ = 1.62, *p*<.05. *Post hoc* analysis of group means showed that subjects receiving TNFα alone differed significantly from those that received vehicle alone, as well as those who received fluorocitrate prior to TNFα treatment, *p*<.05. No other differences approached significance, *p*>.05. This experiment demonstrates the necessity for glial metabolism in order for TNFα treatment to produce a long-term spinal learning deficit, and provides evidence that exogenous TNFα treatment may lead to sustained glial release of endogenous TNFα.

### TNFα inhibition prior to testing reverses the inhibition of spinal learning induced by glial activation

The previous experiments have shown TNFα to be sufficient to produce a long-term inhibition of spinal learning, and that glial activation is necessary to produce this effect. Lipopolysaccharide (LPS) is a bacterially-derived endotoxin that is most often used to experimentally challenge the immune system. LPS binds the toll-like receptor 4 (TLR4) on macrophages (and microglia) activating these cells. The LPS-glial interaction produces a host of biological consequences: inducing histamine release, vasodilation, activation of blood coagulating factors, as well as the release of a host of inflammatory cytokines, including TNF [37]. Prior work has also shown that LPS is sufficient to undermine spinal learning [38]. Despite the varied effects of LPS, the present experiment was designed to test the specific contribution of LPS-induced glial release of TNF by inhibiting TNF activity prior to LPS administration, in order to further investigate the natural interaction between glial activation, TNFα release, and the expression of the spinal learning deficit.

Twenty-four hours after complete transection, subjects (n = 6 per group) were given an intrathecal injection of either 100 µg LPS or vehicle. This dose of LPS has been used previously to induce a spinal learning deficit [38]. Twenty-four hours later, subjects were administered an intrathecal injection of either sTNFR1 (700 ng) or vehicle. All subjects were then tested for instrumental learning.

**Figure 9:**
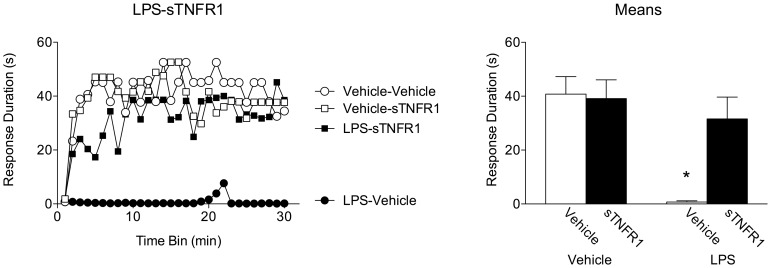
Glial activation generates a lasting inhibition of spinal learning that is reversed by TNFα inhibition. Subjects received an intrathecal injection of the potent glial activator lipopolysaccharide (LPS; 100 µg) or vehicle. Twenty-four hours later, subjects received either sTNFR1 (700 ng) or vehicle, followed by testing for instrumental learning. Left panel depicts response duration over time. LPS treatment alone caused a deficit in spinal learning, while sTNFR1 treatment after LPS allowed for increased response durations over time. Right panel shows group response durations collapsed over time. Subjects that received LPS alone differed significantly from all other groups. Shaded areas represent SEM over time; error bars indicate SEM for group means. Asterisk signifies statistical significance, *p*<.05.

Subjects that received sTNFR1 or vehicle alone were able to learn (Figure 9). As expected, subjects receiving LPS alone exhibited a robust learning deficit. Interestingly, subjects that received sTNFR1 24 hours after LPS treatment were able to learn. An ANOVA revealed main effects of LPS treatment [F_(1, 28)_ = 12.61], sTNFR1 treatment [F_(1, 28)_ = 4.74, and time [F_(29, 812)_ = 4.97], *p*<.05. Likewise, there were significant LPS X sTNFR1 [F_(1, 28)_ = 5.88] and Time X LPS interactions [F_(29, 812)_ = 1.82], *p*<.05. *Post hoc* analysis of group means confirmed that the group receiving LPS alone was significantly different than all others, *p*<.05. No other effects were significant, *p*>.05. As LPS has been shown to induce glial activation and glial release of TNFα, this finding suggests that LPS-induced inhibition of spinal learning may be mediated by glial TNFα. The finding that TNFα inhibition blocked the expression of the spinal learning deficit when given 24 hours after LPS treatment also lends further evidence to the notion that TNFα undermines adaptive plasticity through sustained activity, rather than inducing an immutable change in neural functioning or synaptic strength.

### Inhibition of spinal learning engages calcium-permeable AMPA receptors

Having shown TNFα to be a necessary and sufficient component in the inhibition of spinal learning, we were also interested in how TNFα may engage downstream processes to manifest this behavioral effect. Previous research has shown that neuronal TNFα receptor activation causes an increase in membrane trafficking of GluR2-lacking AMPA receptors [39]. Unlike AMPA receptors that express the GluR2 subunit, these receptors are calcium-permeable. This permeability allows for a substantial increase in postsynaptic excitability, and with sufficient stimulation, can lead to excitotoxicity. This phenomenon has recently been shown to underlie cell death following SCI [6]. The current experiment assessed whether the spinal learning deficit (induced by either intermittent stimulation or TNFα treatment) engages calcium-permeable AMPA receptors.

Twenty-four hours after complete transection, subjects (n = 10 per group) were given one of three initial treatments: either an intrathecal injection of TNFα (6000 pg), intrathecal injection of saline vehicle, or 6 minutes of intermittent stimulation. Twenty-four hours later, all subjects were given a secondary treatment: intrathecal injection of either the GluR2-lacking AMPA receptor antagonist Naspm (10 mM) or vehicle. Naspm at this dose has been shown to attenuate ischemia-induced cell death in the hippocampus [29], [30]. All subjects were then tested for instrumental learning. This design allowed for vehicle control subjects to be compared to both TNFα -treated and stimulated experimental groups. As such, these comparisons were analyzed separately.

**Figure 10:**
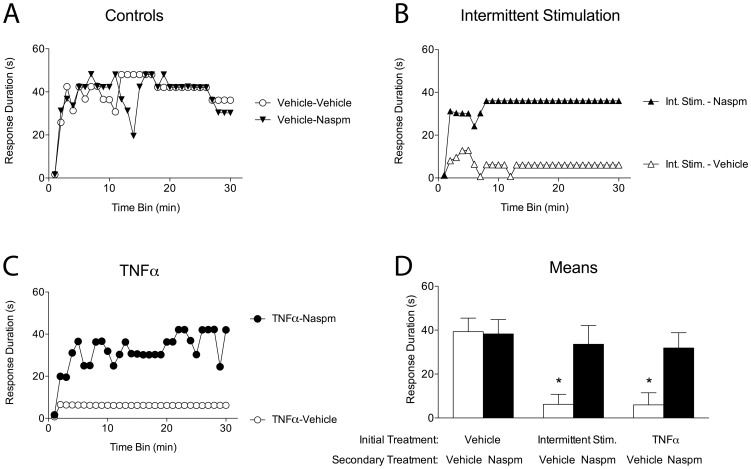
Stimulus- and TNFα -induced inhibition of spinal learning requires calcium-permeable AMPA receptor activity. Subjects received an initial treatment of vehicle, TNFα, or intermittent shock, followed 24 hours later by a secondary treatment of either vehicle or the calcium-permeable AMPA receptor antagonist Naspm. All subjects were then tested for spinal instrumental learning. A) Control Groups. Subjects that received initial vehicle injections were able to learn, regardless of secondary treatment. B) Intermittent Stimulation. Initial treatment with intermittent stimulation produced a learning deficit in those subjects that received vehicle as their secondary treatment. In contrast, those that received Naspm were able to learn significantly better than those that received vehicle. C) TNFα. Initial treatment with TNFα produced a learning deficit in those subjects that received vehicle as their secondary treatment. In contrast, those that received Naspm were able to learn significantly better than those that received vehicle. D) Group means, collapsed across time. Shaded areas represent SEM over time; error bars indicate SEM for group means. Asterisk signifies statistical significance, *p*<.05.

As expected, those subjects that received either vehicle or Naspm alone were able to learn (Figure 10A). Likewise, stimulation-treated subjects that received a secondary vehicle treatment failed to learn (Figure 10B). Interestingly, those that received Naspm after intermittent stimulation did not exhibit a learning deficit. When comparing these groups, ANOVA revealed main effects of intermittent stimulation treatment [F_(1, 36)_ = 7.39], and time [F_(29, 1015)_ = 5.89], *p*<.05. There was also a significant Stimulation X Naspm interaction [F_(1, 36)_ = 4.16] and Time X Stimulation interaction, F_(29, 1044)_ = 1.60, *p*<.05. *Post hoc* analysis of group means confirmed that the stimulation-treated group that received secondary vehicle treatment differed significantly from the other groups, *p*<.05.

Similar to the stimulation-treated subjects, those that received an initial TNFα treatment followed by vehicle were unable to learn, while those that received Naspm after TNFα treatment did not exhibit a learning deficit (Figure 10C). When comparing these two groups to vehicle controls, an ANOVA revealed main effects of TNFα treatment [F_(1, 36)_ = 8.98] and time [F_(29, 1044)_ = 5.28] and a significant interaction between the two [F_(29, 1044)_ = 1.66], *p*<.05. There was also a marginally significant interaction between TNFα and Naspm treatment, F_(1,36)_ = 4.08, *p* = .05. *Post hoc* analysis of group means confirmed that the TNFα -treated group that received secondary vehicle treatment performed worse than Naspm-treated, or double-vehicle groups, *p*<.05. These findings suggest a necessary role for calcium-permeable AMPA receptors in the inhibition of spinal learning, and suggest a common mechanism of action between intermittent stimulation and TNFα in producing this effect.

## Discussion

The present findings demonstrate that TNFα is necessary and sufficient for generating lasting inhibition of spinal learning in our instrumental learning paradigm. We found that the long-term inhibition of spinal learning induced by TNFα administration required glial metabolism, and could be reversed by a TNFα inhibitor given prior to testing. Taken together, these findings suggest a critical role for glial TNFα in undermining spinal learning, and demonstrate the therapeutic potential for TNFα inhibitors in restoring the capacity for adaptive spinal plasticity.

We used a simple model of spinal instrumental learning to assess the effect of TNFα on spinal plasticity. This model is unique in that it allows for pharmacological and physiological manipulations of the isolated spinal cord, while providing a behavioral measure of spinal plasticity. Further, the findings from this paradigm translate well to more naturalistic SCI preparations such as recovery in spinal cord contusion and stepping in chronic transection, providing clinical relevance to these pursuits [13], [17], [40].

One concern with testing the role of TNFα in spinal instrumental learning is that the transection injury used in this model could potentially alter baseline TNFα levels in the essential spinal circuitry where spinal learning takes place. Studies of the expression profile of TNFα following spinal cord injury have shown that TNFα peaks around 1 hour after injury, and then slowly fades over the next 48–72 hours [41]. Given these findings, and the fact that subjects in the current experiments were allowed to recover for 24 hours following complete transection, it is likely that any injury-induced changes in TNFα expression caudal to the transection site have returned to baseline by the time of testing. Further, having shown that unstimulated control subjects are able to exhibit spinal instrumental learning, the TNF levels in these subjects is clearly too low to affect the acquisition of this learning response.

### Metaplasticity in the Spinal Cord

In 1982, Cooper and colleagues introduced a mathematical theory to describe how synaptic strength is informed by prior experience (BCM theory) [42]. They described a “modification threshold” that is raised or lowered as a result of experience, producing an alteration in susceptibility to future plasticity. In effect, this phenomenon describes the plasticity of plasticity, or as later termed, “metaplasticity” [43].

In a series of studies on ocular dominance plasticity, Bear and others demonstrated a behavioral correlate for the theoretical framework laid by Cooper and colleagues [44]. They showed that in response to a period of monocular deprivation, synaptic strength in the contralateral visual cortex was weakened while responses in the ipsilateral visual cortex were strengthened [44], [45]. Thus, prior exposure to monocular deprivation produced a metaplastic effect on future synaptic strength. Others have linked metaplasticity to the release of neuromodulators, such as substance P and 5HT, which have been shown to affect the induction and expression of plasticity in spinal cord interneurons, eliciting metaplastic effects and long-term synaptic reorganization within lamprey locomotor networks [46], [47].

Here, and in previous experiments, we have expanded the view of metaplasticity to include measures of behavioral output from the spinal cord [19]. As in the example of ocular dominance plasticity, prior exposure to a stimulus (intermittent stimulation) produces a lasting change in future plasticity (in this case, an inhibition of spinal learning). It is not clear whether this alteration reflects a shift in modification threshold, or perhaps a biological switch, that inhibits spinal learning. Previous data do suggest though, that this effect does not reflect a general inhibition of spinal function. Indeed, at the same time spinal learning is inhibited, reactivity to mechanical stimulation is enhanced (allodynia) [22]. Intermittent stimulation engages a form of plasticity, the expression of which affects whether a selective response modification (spinal learning) can occur. Similar to the metaplastic effect in ocular dominance, we have shown that the stimulation-induced spinal learning deficit is an active process, modulated by NMDA receptor function [20], [22], [48], [49]. We have shown that treatment with a NMDA receptor antagonist (MK-801) prior to intermittent stimulation will block the induction of the learning deficit [22]. Further, similar to the metaplastic effects seen in the lamprey spinal cord model, we have shown that the stimulation-induced inhibition of spinal learning reflects alterations in group I metabotropic glutamate receptor activation, downstream protein kinase C activity, and requires *de novo* protein synthesis [19], [21], [46], [47]. Importantly, the stimulus-induced impairment in spinal learning is not immutable; the effect of intermittent stimulation decays over time and can be reversed by both behavioral and pharmacological treatments [18], [23], [50]. These observations suggest that intermittent stimulation has a general modulatory effect on plasticity, and as it alters the capacity for the future expression of other forms of plasticity (spinal learning), it can be characterized as a form of spinal metaplasticity.

### Possible Mechanisms of Action

While we have demonstrated that TNFα is necessary and sufficient to produce the inhibition of spinal learning in this paradigm, the question remains as to how TNFα might be exerting this metaplastic effect. Prior work has characterized the spinal learning deficit as reflecting a diffuse overexcitation of spinal neurons, producing a saturation effect that stifles the capacity for future learning [16], [19], [24]. The current findings support this view, as the excitatory effects of TNFα have been widely demonstrated. TNFα receptor (TNFR1) activation has been shown to increase neural excitability by directly, and indirectly, affecting ion channels through a number of distinct intracellular pathways. Using cultured hippocampal slices, Furukawa and Mattson (1998) observed a significant increase in current through L-type calcium channels following long-term incubation with TNFα [51]. This effect was dependent upon TNFR1 activation of the downstream transcription factor nuclear factor-kappa B (NF-κB). TNFα application has also been shown to rapidly enhance currents in tetrodotoxin-resistant Na+ channels, leading to acute mechanical sensitization [52]. Interestingly, this effect was mediated by a TNFR1-dependent phosphorylation of p38 MAPK. Coupled with the necessity for this kinase in the glial release of TNFα, this finding suggests a potentially critical role for p38 MAPK in both the neural and glial effects of TNFα [53].

Despite having some direct effects on excitability through ion channels, TNFα has been more widely characterized by its capacity to indirectly affect synaptic strength by modulating glutamatergic neurotransmission. In 2002, Beattie and colleagues demonstrated that glial TNFα acts to increase trafficking of AMPA receptors to the post-synaptic membrane [8]. As such, TNFα-induced AMPA receptor trafficking can potentiate excitotoxicity [4]. Stellwagen et al. refined the view of this phenomenon by illustrating that TNFR1 acts upon the phosphatidylinositol 3-kinase (PI3K) pathway to mediate the trafficking of calcium-permeable AMPA receptors [39]. Further, TNF-mediated trafficking of calcium-permeable AMPA receptors has recently been shown to undermine neural function following SCI [6]. In the present study, we found that specific antagonism of this receptor type rescued the capacity for spinal learning after both TNFα and intermittent stimulation. As high levels of calcium-permeable AMPA receptor activation are known to promote overexcitation, this finding also supports the idea that metaplastic inhibition of spinal learning may reflect a modulatory effect that resembles saturation. Thus, blocking these receptors appears to return the neural environment to a more quiescent state in which an adaptive modification such as spinal learning can occur.

In contrast to the deleterious effects of TNFα on spinal learning, we have also found a number of *beneficial* effects of training with contingent stimulation. Although intermittent non-contingent (uncontrollable) stimulation can induce a lasting impairment in spinal learning, exposure to either regularly-spaced or response contingent (controllable) stimulation can induce a lasting facilitation of spinal learning [18], [54]. We have recently shown a necessary and sufficient role for brain-derived neurotrophic factor (BDNF) in mediating these positive effects [23], [54]. BDNF treatment can protect against the stimulation-induced spinal learning deficit, as well as rescue learning after the deficit has been induced. Interestingly, TNFα and BDNF have been shown to play opposite roles in synaptic scaling [55]. The opposing effects of these two agents suggests a possible constitutive balance between TNFα and BDNF, and tipping one or the other toward overexpression may be key in shifting spinal metaplasticity between adaptive and maladaptive outcomes.

We demonstrated that intrathecal TNFα treatment is sufficient to produce a spinal learning deficit when given immediately prior to testing, and interestingly, 24 hours prior to testing. As discussed above, TNFα can engage a number of excitatory pathways that could be responsible for the long-term deficit. Interestingly, we found that inhibiting TNFα activity prior to testing blocked the long-term TNFα-induced learning deficit. This finding suggests that sustained TNFα activity is necessary in order for the deficit to be expressed. From this perspective, TNFα can be thought to act in one of two ways: either the exogenous TNFα is continuing to activate TNF receptors 24 hours after administration, or the administered TNFα is inducing the release of endogenous TNFα stores. Though acute TNFα administration has not yet been shown to directly elicit sustained TNF receptor activation, the capacity for TNFα to stimulate the glial production and release of TNFα has been shown [36]. The autocrine function of TNFα can produce a feed-forward loop in which the release of inflammatory mediators can be sustained for long periods of time [56]. Our finding that TNFα protein levels were significantly increased 24 hours after intermittent stimulation supports this viewpoint. Thus, TNFα overexpression (from exogenous injection or elicited by intermittent stimulation) may engage such an autoregulatory signaling loop that perpetuates TNFα activity and generates a long-term metaplastic inhibition of spinal learning. This is a topic of ongoing studies.

### Therapeutic Potential for TNFα inhibition

At normal physiological levels, TNFα has been shown to play an important role in regulating synaptic homeostasis [9]. It is in response to neural insult or immune challenge that TNFα overexpression can occur, causing an inflammatory response that may undermine proper neural functioning. The inhibition of TNFα has recently gained attention as an important tool in fighting a number of inflammatory processes, as a number of TNFα inhibitors (infliximab, etanercept, and adalimumab) are currently indicated for the treatment of arthritis and psoriasis [57]. Further research is expanding the role of TNFα inhibition as a therapy, as it has been shown that selectively ablating TNFα receptors can attenuate dopaminergic neurotoxicity, a major neural consequence believed to underlie the development of Parkinson's disease [58], [59]. While TNFR1 signaling has been characterized as inducing pro-inflammatory effects, the other TNF receptor subtype, TNFR2, has been shown to mediate anti-inflammatory, protective processes after neural insult. Under normal physiological conditions, TNFR1 is constitutively expressed in a number of cell types throughout the spinal cord, while TNFR2 is largely restricted to hematopoietic and endothelial cells [60], [61]. Following spinal cord injury, both receptor types are upregulated in neurons, astrocytes, and oligodendrocytes [62]. While TNFR1 signaling after injury has been implicated in mediating a number of deleterious processes that undermine recovery, it has been suggested that the upregulation of TNFR2 after SCI may reflect a compensatory, protective response [63]. Although current ELISA technology does not differentiate between soluble and membrane-bound forms of TNF, it is most likely that the deleterious effects on spinal learning are due to primarily to activation of the TNFR1 by soluble TNF, as we demonstrate that exogenous soluble TNF is sufficient to undermine spinal learning, and treatment with a soluble TNFR1 (which sequesters and inhibits available TNF) protects against these effects.

After SCI, TNFα expression levels increase rapidly, peaking within 2–6 hours, and slowly returning to baseline across the next 72 hours [41]. Thus, within this window when the spinal microenvironement is extremely vulnerable, the threat of TNF contributing to maladaptive spinal plasticity, as well as excitotoxicity, is high. We have recently shown that blocking TNFα activity with sTNFR1 after spinal contusion injury significantly reduces the trafficking of calcium permeable AMPARs to synaptic membranes, and attenuated exitotoxic cell death [6]. For spinal cord injury in the natural environment, the possibility for TNF overexpression is compounded by the likelihood of concomitant noxious input from other injuries sustained during traumatic spinal cord injury (i.e. lacerations, broken bones, etc; [25]). The intermittent stimulation used in our spinal plasticity paradigm models the peripheral nociceptive input that may accompany a spinal cord injury, and to this end, we have demonstrated that intermittent stimulation after an experimental spinal contusion injury creates a lasting deficit in locomotor recovery [17]. Recent work has demonstrated that TNFα is a key mediator of nociceptive plasticity [11], [12], [64], [65]. The current findings suggest that nociceptive plasticity (induced by intermittent stimulation) is a TNF-mediated process that has a metaplastic effect on future adaptive spinal modifications. As such, therapies that quell the overexpression of TNFα may encourage recovery of function not only by blocking nociceptive plasticity, but by reinstating the capacity for adaptive plasticity. The benefits of promoting adaptive plasticity in the injured spinal cord have been made evident in recent animal studies, as well as a promising human case study [14], [66]. By resolving neurobiological impediments to adaptive plasticity, we can lay the groundwork for an environment that is receptive to positive change, and effective rehabilitation strategies can be realized.
